# Why Are General Moral Values Poor Predictors of Concrete Moral Behavior in Everyday Life? A Conceptual Analysis and Empirical Study

**DOI:** 10.3389/fpsyg.2022.817860

**Published:** 2022-06-30

**Authors:** Tom Gerardus Constantijn van den Berg, Maarten Kroesen, Caspar Gerard Chorus

**Affiliations:** Department of Engineering Systems and Services, Faculty of Technology, Policy and Management, Delft University of Technology, Delft, Netherlands

**Keywords:** moral values, moral decision-making, moral behavior, Moral Foundation Theory, compliance with COVID-19 measures, contextual aspects of moral decision-making, theory of Morality as Cooperation

## Abstract

Within moral psychology, theories focusing on the conceptualization and empirical measurement of people’s morality in terms of general moral values –such as Moral Foundation Theory- (implicitly) assume general moral values to be relevant concepts for the explanation and prediction of behavior in everyday life. However, a solid theoretical and empirical foundation for this idea remains work in progress. In this study we explore this relationship between general moral values and daily life behavior through a conceptual analysis and an empirical study. Our conceptual analysis of the moral value-moral behavior relationship suggests that the effect of a generally endorsed moral value on moral behavior is highly context dependent. It requires the manifestation of several phases of moral decision-making, each influenced by many contextual factors. We expect that this renders the empirical relationship between generic moral values and people’s concrete moral behavior indeterminate. Subsequently, we empirically investigate this relationship in three different studies. We relate two different measures of general moral values -the Moral Foundation Questionnaire and the Morality As Cooperation Questionnaire- to a broad set of self-reported morally relevant daily life behaviors (including adherence to COVID-19 measures and participation in voluntary work). Our empirical results are in line with the expectations derived from our conceptual analysis: the considered general moral values are poor predictors of the selected daily life behaviors. Furthermore, moral values that were tailored to the specific context of the behavior showed to be somewhat stronger predictors. Together with the insights derived from our conceptual analysis, this indicates the relevance of the contextual nature of moral decision-making as a possible explanation for the poor predictive value of general moral values. Our findings suggest that the investigation of morality’s influence on behavior by expressing and measuring it in terms of general moral values may need revision.

## Introduction

Studies focusing on the empirical investigation of people’s general moral values, implicitly or explicitly assume these to be relevant for the explanation and prediction of people’s behavior in everyday life. Theories within contemporary moral psychology that aim at the conceptualization and measurement of people’s general moral values, such as Moral Foundation Theory (MFT) (Haidt and Graham, 2007; Graham et al., 2013) and Morality as Cooperation (MAC) (Curry et al., 2019), typically refer to an evolutionary explanation for the existence and content of moral values. Indeed, this only makes sense when the identified moral values also influence actual behavior. Furthermore, one important reason to study people’s general moral values seems to be its potential influence on decision-making and behavior. Their relevance for (applied) researchers, as well as policy makers, would considerably diminish were it assumed that moral values do not affect nor predict acts performed in everyday life. The relationship between general moral values and the concrete behaviors people perform in everyday life^1^ then seems to be an important underpinning of the empirical study of moral values.

However, despite forming an important assumption of moral value research, much is still unknown about the theoretical as well as the empirical aspects of this relationship. Theoretically, for instance, there is no general agreement on how moral decision-making exactly works and how different presumably relevant phenomena, like moral values, moral judgments, empathy, emotions, intuitions and reasoning etc., interact in moral decision-making and consequent behaviors (Schwartz, 2016; Hoover et al., 2019). Exemplary in this regard is the debate between intuitionists and rationalists on moral reasoning and moral judgment (Bucciarelli et al., 2008; Musschenga, 2009). Intuitionists claim that moral values feature within an emotional and intuitive process of moral judgment formation and regard any deliberate reasoning as *post hoc* confabulation. Conscious reasoning involving moral values and moral principles is thereby virtually excluded from having any direct influence on moral judgment (Haidt, 2001). Rationalists give a more prominent place to the process of conscious reasoning in forming moral judgment, featuring moral values and moral principles (Kohlberg, 1984; Rest, 1986; Kasachkoff and Saltzstein, 2008; Kennett and Fine, 2009).

Empirically, on the other hand, research investigating the link between general moral values and concrete moral behavior in daily life is relatively scarce (Ellemers et al., 2019; O’Grady et al., 2019). As Graham et al. (2012) state, most research on individual differences in morality has concentrated on people’s prioritization of values and less on how these differences influence people’s moral behavior in real life. Likewise, Ellemers et al. (2019) conclude in a major review of psychological studies on morality since 1940 that, although authors commonly express that their main interest in studying a certain aspect of morality lies in the explanation and prediction of moral behavior, the vast majority of studies concentrates on how people *think* about morality instead of how such moral beliefs and attitudes influence actual moral behavior. As Ellemers et al. (2019) state, the assumed relationship between the studied moral constructs and behavior remains thereby mainly hypothetical. In line with these observations, general moral values have been more commonly related to attitudinal variables and other general dispositions (Graham et al., 2011; O’Grady et al., 2019). MFT has, for instance, been extensively related to people’s political ideology (Haidt and Graham, 2007; Graham et al., 2009) and socio-political attitudes (e.g., Kugler et al., 2014; Dickinson et al., 2016). Yet, the question whether someone who scores higher on a moral value scale also shows more moral behavior (related to that dimension) in everyday life has not been satisfactorily answered (Graham et al., 2012; Hoover et al., 2019).

However, this does not mean we are completely in the dark about the empirical aspects of the relationship between general moral values and concrete moral behavior. First, to get a better grasp of this relationship, it is insightful to turn to the more general field of value research, which focuses on the broader concept of basic or personal values (Schwartz, 1992; Sagiv et al., 2017). This field has extensively and more systematically studied the empirical relationship between values and behavior than has so far been done in the moral domain. In particular, the vast amount of literature that builds on Schwartz’s theory of basic values (Schwartz, 1992; Schwartz et al., 2012), typically defining values as desirable *trans*-situational goals or abstract ideals guiding people’s life, has linked values to a broad set of behaviors (e.g., Bardi and Schwartz, 2003; Schwartz and Butenko, 2014; Schwartz et al., 2017). Here, values are usually regarded as important notions to predict and explain how people think, decide, and act within value-relevant situations (Rohan, 2000; Maio et al., 2006; Miles, 2015; Schwartz et al., 2017; Sagiv and Roccas, 2021). However, at the same time, it is acknowledged that empirical studies generally find only weak to moderate effects of values on behavior (Cieciuch, 2017; Lee et al., 2021). Studies which consider the morally relevant basic values of “benevolence” and “universalism”^2^ in connection to moral or pro-social behavior, find similarly sized effects (e.g., Bardi and Schwartz, 2003; Caprara et al., 2012; Miles, 2015).

The generally found weak to moderate effects between values and behavior may even be overestimations. Boyd et al. (2015) note that the self-report measures of behavior in these studies often have a rather general level of abstraction^3^, which risks relating only different aspects of one’s self-concept (one’s values to one’s conception of one’s broad behavioral or personality traits) instead of one’s values to actual concrete behavior. This suggests that the effects of values on actual concrete forms of behavior may be weaker than found in those studies. Such weak associations between general values and concrete behavior are also in line with what has been generally found between general attitudes and more concrete or specific forms of behavior in other fields, like transportation (Kroesen and Chorus, 2018). These findings, at least, serve as an indication of what can be expected of the effect of general *moral* values on concrete *moral* behavior, namely that these may be rather weak.

Secondly, we can look into studies that have related moral values to specific forms of moral behavior. While not specifically focusing on a systematic investigation of the general moral value – moral behavior relationship [though see O’Grady et al. (2019) for a recent more relevant effort with regards to MFT], a number of studies from different fields do take general moral value measures into account (as part of their models) to explain specific forms of morally relevant behavior [e.g., Reynolds and Ceranic, 2007; Tarry and Emler, 2007; Cohen et al., 2014; Nilsson et al., 2016; Vainio and Mäkiniemi, 2016 (*Study 1*); Qian and Yahara, 2020; van den Berg et al., 2020; Presti et al., 2021; Díaz and Cova, 2022]. When inspecting their results, we find, in line with the above, only small effects. While often concluding that the measured general moral values are important predictors of the considered moral behaviors, the reported correlations, when significant, do not exceed the 0.2–0.3 range. This means that at best about 4–9% of the variance in the studied behavior is explained by people’s general moral values. It may even be expected that the actual number of studies finding very weak to no empirical relationships is higher due to publication bias (Rothstein et al., 2005).^4^ (Vainio and Mäkiniemi, 2016, *Study 2*) find somewhat higher effects by making the moral values specific to the context of the behavior that was studied (climate-friendly consumption). This result suggests that contextual factors may play an important role in the influence of morality on behavior, possibly undermining the attractive idea of having a limited set of fundamental moral values that can represent one’s morality and is able to predict behavior in different contexts.

In this study, we want to further contribute to the investigation and understanding of the relationship between general moral values and concrete moral behavior, theoretically as well as empirically. Theoretically, we offer new insights, without solving the difficult debate concerning the exact process of moral decision-making, through a conceptual characterization of the general moral value-concrete moral behavior relationship that should be acceptable for both intuitionists as well as rationalists. To articulate the relationship, we build on and extend a well-established ethical decision-making model (Rest, 1986; Schwartz, 2016). From this conceptual characterization we derive the notion that general moral values are expected to be poor predictors of concrete moral behavior in everyday life. A reason for this is the contextuality^5^ of the moral decision-making process that arises from our analysis. In the subsequent empirical part of the study, we look into the empirical side of the relationship between general moral values and concrete moral behavior. In three separate studies, we relate measures of general moral values to different kinds of self-reported morally relevant behaviors in daily life. In the last study, we also take more specific moral values into account to investigate whether moral values become stronger predictors when they are tailored to the context of the behavior that they are to predict.

### Specifying “General Moral Values” in Line With Moral Foundation Theory and Morality as Cooperation

Before embarking on the conceptual analysis and subsequent empirical investigation, we start by specifying in some more detail what we refer to by the term “general moral values.” With general moral values we mean trans-situational moral ideals which guide our moral judgment, i.e., our judgment in terms of “right” and “wrong.” These *trans*-situational moral ideals are the focus of theories such as Moral Foundation Theory and Morality As Cooperation theory. The extent to which individuals endorse these different moral ideals or general moral values is empirically measured with their accompanying questionnaires. Within these theories, general moral values get the character of general moral dispositions. We will explicate this in a bit more detail in the following.

Considering the MFT, what can be viewed as an individual’s general moral values is one’s endorsement of the five moral foundations (Care/harm, fairness/cheating, loyalty/betrayal, authority/subversion, sanctity/degradation). According to MFT, these universal moral foundations have an evolutionary origin, functioning as fitness enhancing solutions to distinct problems of (group) survival; as such, they exist as innate modules on which every human being’s morality is built. To what extent each moral foundation is developed into an individual’s actual morality depends on factors like cultural influences, upbringing and individual experience. The development of a moral foundation reflects a sensitivity to situations, concepts, principles, beliefs etc. that belong to a certain moral domain, and results in intuitive moral judgments (Graham et al., 2013). The level of development of each moral foundation within an individual’s morality can be measured with the Moral Foundation Questionnaire (MFQ)^6^. The MFQ-items ask about the relevance of several general moral considerations when making a moral judgment (e.g., “whether or not someone suffered emotionally”- care/harm foundation), referred to as the relevance-items, and one’s agreement with several general moral statements (e.g., “I am proud of my country’s history”- loyalty/betrayal foundation), referred to as the judgment-items. Each item belongs to a specific moral foundation. The score per foundation is then taken to be the individual’s level of endorsement of this general moral value.

The Morality as Cooperation theory and questionnaire offer a similar structure as MFT. However, as MAC explicitly starts from the theoretical premise that morality evolved as a biologically and culturally developed set of solutions to problems of *cooperation in social life*, it identifies a different -and theoretically stronger substantiated- set of general moral values. According to MAC, each problem of social cooperation and each solution –which are basically those of zero-sum games– gives rise to a distinct moral domain and an accompanying general moral value. MAC identifies seven of those: family values, group loyalty, reciprocity, bravery, respect, fairness, and property rights (Curry et al., 2019). Again, the development of each moral value differentiates between individuals and can be measured for every individual by a questionnaire -MAC-Q- which has a similar structure as MFQ. It asks about the relevance of general moral considerations for the respondent and about the respondents’ level of agreement with general moral statements.

In sum, what can be regarded as people’s general moral values in light of these empirical moral value theories, may be described as fairly stable psychological dispositions that latently exist within the individual. Also described as moral traits (Haidt and Joseph, 2007), they reflect a *trans*-situational sensitivity to a moral domain, influencing decision-making across contexts. Accordingly, these general moral values can be measured outside of a specific contextual situation by asking about general moral considerations and principles.

Identifying such moral dispositions, is of course particularly valuable when these have an effect on behavior; this would help enable the prediction –and possibly also the influencing– of behavioral patterns. As such, it would, for instance, greatly contribute to MFT’s claim to “pragmatic validity” (Graham et al., 2011, 2013), a validity of the theory based on the extent to which it produces further and new forms of understanding of morality and behavior, especially with regards to people’s actual behavior within everyday life (Rozin, 2006). In the following, a conceptual characterization of the general moral value-concrete moral behavior relationship is explicated to investigate whether such a relationship is to be expected on theoretical grounds.

### A Minimal Characterization of the General Moral Value–Concrete Moral Behavior Relationship

For a general moral value, considered as an individual moral disposition, to perform as a predictor of concrete moral behavior, its presence within an individual would have to (regularly) lead to this behavior. To get a better understanding of the possible effect of someone’s general moral values on one’s behavior in daily life, it is fruitful to take the perspective of an individual making a moral decision. Even though there is not a universally accepted theory of how individual moral decision-making exactly works, nor how moral values exactly feature within it, it does seem possible to identify basic elements within this process that at a minimum need to become manifest before one’s general moral value, regarded as an individual moral disposition, can have an effect on actual behavior. Rest’s (1986) basic Four Component Model of ethical decision-making is a useful starting point here. Rest (1994, p. 23) states that his Four Component Model is an answer to the question: “What must we suppose happens psychologically in order for moral behavior to take place?.” As an answer, the model depicts four different process stages an individual has to go through in order to make a moral decision and perform the corresponding moral behavior: *moral awareness, moral judgment, moral intention*, and *moral behavior*.

Though initially developed as a rational model of moral decision-making, a recent extension of the model by Schwartz (2016) shows that, besides rational, it can also accommodate intuitive/emotional and mixed conceptions of moral decision-making. As it can incorporate different conceptions of moral decision-making, we can apply the model to answer the question that is relevant to our study, posed in the same vein as Rest: “At a minimum, what processes need to take place before an individual’s endorsed general moral value can affect one’s behavior?.” Although the four-component model provides only a minimal characterization of the general moral value-concrete moral behavior relationship, it does give insight into what can be expected from the empirical prediction of concrete moral behavior from general moral values and why. In short, it brings forward that this relationship can be expected to be indeterminate due to the contextuality of the moral decision-making process. In the following, this is explicated by discussing the four stages that an individual’s generally endorsed moral value needs go through within a specific decision situation before it can have an effect on behavior.

### Four Necessary Process Stages for Linking General Moral Values to Concrete Moral Behavior

#### Moral Awareness

To start with, for someone’s general moral value to become effective in decision-making at all, a person needs to become *aware*^7^ that within a certain concrete decision situation this moral value plays a role. Overlooking the relevance of an endorsed moral value should not be considered as a rare exception. First of all, social situations need to be interpreted and these can be ambiguous to the decision maker (Latane and Darley, 1970). Is a girl screaming from laughter and fun as she is being teased by friends or from fear as she is harassed by bullies (Thornberg et al., 2018)? Secondly, the decision maker needs to become aware of the actions possible in the situation and of their factual consequences and effects on others. For instance, one may simply not realize that an action breaks a promise or that it has certain harmful consequences for a (group of) person(s). It may be part of one’s customary behavior to which no further (critical) thought is given. Also, some behaviors or situations, though relevant to one’s endorsed general moral value, may be less typical exemplars or instantiations of this value and therefore not as easily linked to it by the individual (Hanel et al., 2017). Third, several psychological processes, which Bandura et al. (1996) grouped under the name of processes of “moral disengagement,” make it possible that even when one realizes the factual effects of an action or situation, the *moral significance* of it may still not be recognized. For instance, one may not really empathize with victims due to blaming the victim for the harm suffered. Or, due to processes of dehumanization, one may not recognize [or (sub-)consciously downplay] the moral worth of victims (e.g., looking at people from an out-group as being “inferior”) and thereby not become fully aware that a generally endorsed moral value applies to them (Kelman, 1976; Haslam and Loughnan, 2014). Haslam (2006) emphasizes that this is not restricted to contexts of extreme violence but is actually an everyday social phenomenon. Another important aspect of realizing the moral significance of a situation is that it is morally significant to *you* – i.e., that one feels morally responsible to do something. A person may simply not have reflected thoroughly on one’s own role in a situation, or, as Bandura et al. (1996) explains, diffuse the moral responsibility to others to evade interference. A generally endorsed moral value will in such cases not influence further decision-making and action.

Psychological research shows that there are many situational and individual factors that can influence these different rounds of interpretation (O’Fallon and Butterfield, 2005; Craft, 2013; Schwartz, 2016). This then has the result that a generally endorsed moral value, even when it could be relevant within a specific situation, sometimes may not start to play a role in the decision-making process of the individual, causing a first indeterminacy in the empirical general moral value-concrete moral behavior relationship.

#### Moral Judgment

Once an individual has become aware of the possible lines of actions within a decision situation, of their moral significance, and of one’s responsibility, he or she needs to *morally judge* which line of action is the right one. An indeterminacy in the prediction of behavior from general moral values can arise in this stage due to the fact that in real life a moral decision situation often consists in weighing competing moral values against each other, whether through a deliberative, intuitive, or mixed process. This competition between moral values is not taken into account in a standard moral value questionnaire like MFQ, i.e., one can indicate to find all values (just as) important (Frimer and Walker, 2008). Furthermore, given that some situations are more morally salient than others (Jones, 1991), it is conceivable that in one situation a moral value is more salient than in another. The idea that the relative importance of moral values changes across contexts is also in line with social psychological theories such as Social Identity Theory (Tajfel and Turner, 1979) and Self-Categorization Theory (Turner et al., 1987, 2006), which claim that people can identify themselves differently in different contexts, influencing the importance of values that are partially constitutive of such identities. This makes weighing different lines of actions, informed by underlying moral values, a highly contextual endeavor. To make this more tangible, think of a civil servant who generally prefers the moral value of fairness over loyalty. It may be expected that this person declines a favor asked by a friend to issue a permit without due procedure. However, this does not mean that, when this friend and a stranger are drowning and he can only save one, he will toss a coin, or use any other fair procedure, to determine who to save.

A second indeterminacy in predicting behavior from general moral values arises within this stage, as the same general moral value may indicate more than one line of action as the right one. In the application of one’s general moral value there is simply not one specific action that it determines. This can lead different persons to different behaviors in the same kind of decision-making context, while claiming to endorse the same general moral value. It can even lead the same person to different behaviors in (only slightly) different contexts. Regarding the first kind of indeterminacy, think of the moral value of care that inclines one person to speed one’s mom to the hospital when she is in an emergency, while it may incline another person in the same kind of situation to stick to the speed limit, in order to not risk injuring others. Regarding the second kind, consider that in a subsequent similar emergency situation, the first person may now stick to the speed limit, remembering that he or she almost caused an accident the last time (this reconsideration being induced by the same general moral value of caring for others). The more general problem seems to be here that when measuring a general moral value, it is not clear what specific meanings or instantiations of this general value is considered by respondents (Hanel et al., 2017). These diverting specific meanings can lead to contradictory behaviors between individuals. Also, the meaning given to the value may be linked to a specific context and, therefore, the measured “general endorsement” by the individual may not be transferable across contexts. This leads to indeterminacy when predicting behavior from general moral value measures.

#### Moral Intention

When an individual judges which action is the morally right one in light of an endorsed moral value, this does not automatically mean that one will also form the *moral intention* to follow through on one’s judgment. Besides moral values, also self-serving values play a role in decision-making, like advancing personal goals and desires. Indeed, moral values are often considered as controlling factors that keep people from only pursuing their short-term selfish desires (Hofmann et al., 2018).

To what extent a moral judgment, based on a moral value, controls for more selfish tendencies and thus to what extent an individual actually sticks to one’s moral judgment in a specific decision situation is influenced by many situational and individual factors. Research shows that the social context, like the presence of peers (Warr, 2002), authority figures (Milgram, 1974), or simply being in a hurry (Darley and Batson, 1973) can have a strong influence on whether someone sticks to generally endorsed moral values and corresponding judgments. Also, following Fishbein and Ajzen’s (1975) *theory of reasoned action*, people are inclined to follow the subjectively perceived prescriptive norms within a given context. These so-called “subjective norms,” defined as a person’s perception of others’ expectations and approval of one’s behavior, can be in line with, but may also go against one’s considered general moral values and corresponding judgments. This imposes the normative pressure to deviate from them in one’s behavior. Individually, people differ in terms of strength of will (May and Holton, 2012), moral courage (Lachman, 2007) and the extent to which moral values and principles are part of their self-identity (Aquino and Reed, 2002; Schlenker et al., 2009), thereby differing in their capacity to “cling to those values even when faced with pressures to act otherwise.” (Schwartz, 2016, p. 761). What becomes clear, is that the extent to which the individual will stick to a generally endorsed moral value in the context of a certain decision situation is quite hard to predict when only relying on a measure of the general endorsement itself. This further contributes to indeterminacy in predicting behavior from general moral values.

#### Moral Behavior

Then finally, even in the case a moral intention is established to act upon one’s moral judgment, there is still one step to take in order for someone’s generally endorsed moral value to affect moral behavior: actually performing the behavior itself. Bringing an intention into action involves “working around impediments and unexpected difficulties, overcoming fatigue and frustration, resisting allurements and keeping sight of the eventual goal” (Rest, 1986, p. 15). From this description it becomes clear that many individual- as well as situational factors can play a considerable role. Examples of the first are perseverance and focus to stick to a decision once it is made, especially in long term projects. Situationally, the circumstances can turn out in many degrees of difficulty and complexity, up to the point that they make it just impossible to engage in the intended moral behavior. In this last step then, from moral intention to moral behavior, there is still ample possibility for the effect of a generally endorsed moral value on behavior to perish just before the finish line.

It should be noted, of course, that what appears here in a sequential order and in a demarcated and deliberate fashion can in reality be an intuitive, non-sequential and rather dynamic process of mutual adjustment. Moral awareness and moral judgment may often virtually arise at the same time. Research on basic values and behavior indicates that the value-behavior causal relationship can go both ways (Maio et al., 2006; Maio, 2016). Also, theories like cognitive dissonance (Festinger, 1957) make clear that someone’s moral judgment and moral value can be adjusted as an effect of an already established intention or performed moral behavior, instead of the other way around. A vast amount of research on rationalization and neutralization techniques (Sykes and Matza, 1957) shows that people can easily (morally) justify self-serving intentions and behaviors of which they somehow know that these are not (totally) right (e.g., Mckercher et al., 2008; Harris and Daunt, 2011; Johnston and Kilty, 2016). Even initial moral awareness may be rationalized away retroactively by, in second instance, blaming the victim, inspired by self-serving considerations that shape someone’s intention. Moral awareness, moral judgment and intention may in this way come up simultaneously, mutually adjusting each other. The potential effect of a general moral value on behavior can thereby be retroactively annulled or never start off, and the direction of the effect may even be reversed within a specific context of decision-making, possibly further contributing to the indeterminacy of predicting behavior from generally endorsed moral values. Furthermore, much of the described process may occur intuitively, subconsciously, and within the blink of an eye (although some moral choices can involve long conscious deliberations).^8^

Now what can we conclude from this conceptual exposition of the general moral value–concrete moral behavior relationship? Despite the complexities and the substantial gaps in our knowledge of the moral decision-making process, it has proven insightful to conceptually break down the process of moral decision-making into the described elements and apply it to the general moral value-concrete moral behavior relationship. This minimal characterization of the relationship shows that someone’s generally endorsed moral value needs to go through all four described phases when one enters a certain moral decision situation, before it can have an effect on actual moral behavior. The outcome of each of these four phases is influenced by many different contextual factors, which render the effect of a general moral value on moral behavior indeterminate. From this analysis we may expect that general moral values are not strong predictors of concrete moral behavior. In fact, it may be expected that no or only weak effects are found. Furthermore, the above suggests that when moral values are more specified to the context of the behavior that they are to predict, this may lead to stronger effects. It can, namely, be expected that these more concrete and specific measures hold more accurate information for a given context on what is considered morally important, i.e., whether one will become aware of this more specific value when it is at stake, or how it holds up against other moral and egoistic values.

In the following, we investigate the empirical relationship between general moral values and concrete moral behavior in everyday life in three empirical studies. In study 1, we study the relationship between the moral foundations of MFT and participation in voluntary work and informal care. In Study 2, we relate four general moral values provided by MAC to compliance with the nationally proclaimed COVID-19 measures during the ‘intelligent’ lockdown in The Netherlands in the period of March–May 2020. Finally, in Study 3, we look into the effects of general moral foundations on the consumption of meat. Additionally, we compare these to the effects of more specific values -in terms of animal welfare- on meat consumption.

## Materials and Methods

### Description of the Used Data Sets

For the three conducted empirical studies, we made use of three different datasets. For Study 1 and 3, we used existing data by combining measurements from various surveys that have previously been administered among members of the Longitudinal Internet Studies for the Social sciences (LISS) panel -a panel conducted by CentERdata (Tilburg University, The Netherlands). For study 2, we collected our own data. The level of generalization that we aim to make on the basis of this data is that of the Dutch population.

To start with the LISS panel data, this is a representative sample of Dutch individuals who participate in monthly Internet surveys for academic research purposes. The panel is based on a true probability sample of households drawn from the population register. Households that cannot otherwise participate are provided with a computer and Internet connection. Given the data collection procedure, the LISS panel (as a whole) is representative of the Dutch population. Table 1 presents an overview of the surveys that were combined for Study 1 and Study 3. The surveys were conducted during the end of 2012 and beginning of 2013. The combinations of the surveys yielded different sample sizes for both studies (presented in the final row).^9^

**TABLE 1:** Overview of surveys and participants across studies 1, 2, and 3.

				Study		
LISS panel survey name	Data collection period	Response (response rate)	Measurements	1	2	3
Social Integration and Leisure, wave 6	February 2013	5,676 (86.0%)	Voluntary behavior (three items), providing informal care (one item)	•		
Consumption decisions and perceptions of animal welfare – Part 2	November 2012	2,648 (87.2%)	MFQ (six items), animal-specific MFQ (six items)	•		•
Consumption decisions and perceptions of animal welfare – Part 1	October 2012	3,038 (79.2%)	Consumption of meat (one item) and meat replacement products (one item)			•
**Own data collection -** Compliance to Corona measures 2020	May 2020	N.A.	MAC (12 items), Compliance to Corona measures (10 items)		•	
Sample size (*N*)				2,320	1,396	2,379

A possible drawback of selecting measurements from multiple surveys is that self-selection biases which may already be present for any individual survey become propagated across multiple surveys. A comparison of the distributions of three socio-demographic characteristics (gender, age and education level) of the different samples with the respective distributions of all LISS panel participants as well as with the Dutch population in 2012, shows that this risk may have indeed manifested itself: especially women and older people are overrepresented, compared to the Dutch population (Table 2). The distribution in terms of level of education is, however, well aligned with the population distribution.

**TABLE 2:** The sample distributions of social-demographic characteristics in comparison to LISS panel and population distributions.

Variable	Categories	Study			LISS panel	Dutch pop. (2012)	Dutch pop. (2019)
		1	2	3			
Gender (%)	Male	29	49.6	29	49	49	50
	Female	71	49.9	71	51	51	50
	Other	–	0.5	–	–	–	–
Age (%)	15–24 years	2	66	2	16	15	15
	25–34 years	8	10	9	13	15	15
	35–44 years	16	2	16	16	17	14
	45–54 years	20	10	20	18	18	17
	55–64 years	25	11	25	18	16	16
	65 years and older	28	2	28	20	19	23
Level of education (%)	Lower education	60	7	59	64	63	59
	Higher education	41	93	41	36	38	41
**Sample size (*N*)**		2,320	1,396	2,379			

While the bias in terms of gender and age may affect the mean values of the dependent variables in the considered analysis (if these variables significantly influence the dependent variables) there is no reason to expect that the bias will have (large) effects on the estimated relationships between the considered moral values and behaviors. In our study we are explicitly interested in the latter, not the former.

Study 2 is based on a convenience sample, collected by students in the context of a bachelor course running in May 2020 (*N* = 1,396).^10^ This gave us the opportunity to investigate a very topical moral behavior that virtually everyone had to deal with in the prior months and that is therefore fresh on people’s minds: people’s behavior in relation to nationally proclaimed measures concerning hygiene and social distancing during the ‘intelligent’ lockdown period (March–May, 2020) of the Corona crisis in The Netherlands. Although it enabled us to collect data relatively fast and thus study a topical phenomenon in a timely fashion, using a convenience sample also runs the high risk of not being representative of the population. As Table 2 shows, comparing our sample to the distributions of the Dutch population in 2019 makes clear that it is indeed biased toward higher educated people and persons in the age group of 15–24. For the same reason as above, however, we believe that the consequences of this bias are limited.

For all three studies, the ethical standards with regards to data collection were met. Informed consent was obtained from all participants. Furthermore, the protocol of Study 2 was accepted by The Human Research Ethics Committee of the university to which we are affiliated. The data collection procedure, conducted by CentERdata, collecting the data for study 1 and 3, abided by the European General Data Protection Regulation (GDPR).

### Dependent Variables

When relating moral measures to moral behavior, a point of discussion is how to determine what counts as “moral behavior.” Often the researcher chooses certain forms of behavior that one regards as clearly morally relevant. However, the researcher may be wrong, in the sense that his or her interpretation of this behavior as “moral” does not align with that of the respondents (Meindl and Graham, 2014). Not everyone agrees on what behavior is actually morally relevant. For instance, some may think that choosing one’s mode of transport is a morally relevant choice as it influences one’s impact on the environment, while others do not entertain any moral considerations when deciding whether to take the bike, bus, train, or car. One option that is suggested to tackle this problem is to define moral behavior from a first-person perspective, that is, to let the respondents themselves decide and report when their behavior is morally relevant. This option, however, also has a considerable downside. It is vulnerable to socially desirable answers as well as to widespread psychological mechanisms that downplay the moral significance that a subject attributes to their behavior, such as cognitive dissonance and moral disengagement (Frimer and Walker, 2008). This can lead respondents to not report behavior as morally relevant, while it clearly is, also according to the general standards of the respondent. As the awareness stage of moral decision-making is an important part of our conceptual model, we find it necessary to include this phase and its possible moral evading mechanisms in our measurements. This information would be missed when we only include behaviors in our models of which the respondents report to have become morally aware. Therefore, we selected as dependent variables a broad set of different daily life behaviors that can all be considered as morally relevant, i.e., where moral considerations can and arguably should play a role. It is for this latter feature that they were deemed suitable for and included in the studies.

Furthermore, as we have noted throughout the paper, we focus in this study on *concrete* moral behaviors. This is to clearly measure distinct phenomena, namely people’s general (and specific) moral beliefs and their performed moral behavior, and not run the risk, described above in reference to Boyd et al. (2015), of plausibly tapping into overlapping sources. By concrete moral behaviors we mean that they have a certain level of specificity, i.e., their description takes the form of concrete acts that are part of certain episodes of behavior that can be remembered and recounted as such, rather than the form of a general tendency or trait. Compare in this regard “donating to charity” (Nilsson et al., 2016) to “I try to help others” (Caprara et al., 2012), where the first kind of description falls within our scope and the latter doesn’t. At the same time, we are not aiming at measuring a single behavioral decision by a person. Though concrete, all constructs do reflect behavior over a certain period of time and thus a behavioral pattern. Even participation in a voluntary organization can usually be assumed to be more than just a one-time action.

In Study 1, we focused on participation in voluntary work and providing informal care. As this involves providing help to others without (a large) personal gain, this behavior has a clear moral component. Participation in voluntary work was operationalized as a dichotomous variable, indicating whether the respondent reported to have performed voluntary work for organizations within one of the following fields: human rights, environmental, and religious (yes/no) (Table 3). Providing informal care was operationalized as the number of hours during which the respondent reported to have provided informal care per week on average, in the last 12 months (coded in six categories, see Table 3).

**TABLE 3:** Descriptive statistics of the predicted morally relevant behaviors.

Concept	Question and categories	
	Whether one has participated in voluntary work for one of the following kinds of organizations: organization for humanitarian aid, human rights, minorities, migrants, environmental protection, peace, animal rights, religious, and/or church	
Voluntary behavior (Study 1)	0 = no (%)	92
	1 = yes (%)	8
Providing informal care (Study 1)	How many hours of informal care did you provide per week, on average, in the last 12 months?	
	0 = 0 (%)	74
	1 = 1–8 (%)	18
	2 = 9–16 (%)	4
	3 = 17–24 (%)	1
	4 = 25–32 (%)	1
	5 = 33 or more (%)	1
	Can you please indicate to what extent you comply with the following measures of the RIVM? (1–5) mean (*SD*)	
Adherence to Corona measures (Study 2)	Personal hygiene	3.05 (0.91)
	Not visit the vulnerable	3.8 (1.29)
	Social distancing	3.69 (0.83)
Consumption of meat (Study 3)	Over the last 4 weeks (28 days), on how many days did you eat chicken meat? mean (*SD*)	5.5 (4.3)
Consumption of meat replacement products (Study 3)	Do you ever eat meat replacement products? Meat replacements products include vegetarian balls or burgers, tofu, soy, tempé, or quorn.	
	1 = Never (%)	51
	2 = Tasted it once (%)	14
	3 = Less than 1 time per month (%)	12
	4 = 1 time per month or more often, but less than 1 time per week (%)	12
	5 = 1 – 2 times per week (%)	8
	6 = 3 – 4 times per week (%)	2
	7 = 5 times per week or more often (%)	1

In Study 2, the dependent variable consisted of compliance to the measures proclaimed by The Dutch government during the “intelligent” lockdown of the Corona crisis March–May 2020. As the measures were meant and presented as an important way to reduce hospitalization and to save lives, we regard it as morally relevant behavior. Compliance to the Corona measures was operationalized by using items from a national survey, measuring compliance rates with the proclaimed measures conducted by the National Institute of Public Health (RIVM)^11^. We measured on a 5-point Likert scale (never-always) to what extent people self-reported to comply with certain rules, like washing hands and keeping 1.5 m distance from others. In order to bring down the number of models to be estimated, a Principal Component Analysis (PCA) was conducted to summarize the data.^12^ This revealed that 9 out of 10 items converged on three distinct components that were logically interpretable: compliance with measures involving personal hygiene, not visiting the most vulnerable groups in society, and general social distancing (Table 4). The one item that did not sufficiently load on any of the three components, asking about coughing and sneezing in the elbow, was left out of the analyses. Regarding reliability of the found components, the social distancing component is with a Cronbach’s Alpha of 0.61 just below the 0.7 reliability threshold. As we are here not assuming a latently existing construct, but rather aim to summarize the data in the best way possible (i.e., reduce the data with the least loss of variation), we decided to follow the result given by the PCA and accept the slightly lower reliability for this component. The other components are above the 0.7 reliability threshold (Table 4). For each component the sumscore was computed and included in the analyses.

**TABLE 4:** Rotated component matrix of complying to Corona measures items (Study 2).

Questions and items	Component loadings on dimensions		
Can you please indicate to what extent you comply with the following measures of the RIVM? (1 = never to 5 = always)	Personal hygiene (α = 0.703)	Not visit the vulnerable (α = 0.850)	Social distancing (α = 0.609)
Wash hands often enough	**0.841**	0.039	0.164
Frequently wash hands (more than ten times a day)	**0.835**	0.024	0.020
Wash hands thoroughly (at least 20 s)	**0.715**	0.100	0.085
Use paper towels	**0.512**	–0.064	0.250
Do not visit persons older than 70 years old	0.068	**0.923**	0.094
Do not visit persons with ill health	–0.001	**0.913**	0.169
Not shake hands	0.012	0.140	**0.708**
Keep at a sufficient distance from other people (at least 1.5 m)	0.203	–0.032	**0.754**
Do not have more than three people visiting	0.218	0.234	**0.717**

*Varimax rotation was used to get a simple structure. Number of components extracted was determined based on the component’s eigenvalues, where the eigenvalue of 1 was used as the cut-off value. Loadings in bold signify the item’s selection for the particular component.*

In Study 3, we used the consumption of meat and meat replacement products as dependent variables. Studies show that the consumption of meat is regarded as morally relevant behavior by part of the consumer population (Mäkiniemi et al., 2011). The choice to eat less or no meat has also been extensively linked to moral considerations like animal welfare and its ecological impact (Ruby, 2012). (Not) eating meat and eating meat replacement products are therefore considered as behaviors that have a moral component. The consumption of meat was measured in terms of the number of days the respondent reported to have consumed chicken meat during the last 4 weeks; consumption of meat replacement products was measured on a 7-point Likert scale ranging from never to five times per week or more (Table 3).

### Independent Variables

Since we aim at a general investigation of the effects of general moral values on concrete moral behavior, we made use of two different pre-established scales to operationalize people’s general moral values. The Moral Foundation Questionnaire (MFQ) (see footnote 6; Graham et al., 2011) was used in Study 1 and 3. The Morality as Cooperation Questionnaire (MAC-Q) (Curry et al., 2019) was used in Study 2.

Moral Foundation Questionnaire has been widely deployed to measure people’s general moral values and has been validated across different samples. However, evidence on the structure of the scale does not seem to be fully conclusive. Some studies find evidence for the proposed five-factor structure, though the fit is not always optimal (e.g., Bobbio et al., 2011; Graham et al., 2011; Davies et al., 2014; Nilsson and Erlandsson, 2015; Yilmaz et al., 2016). It has also been suggested that the five-factor structure is further reducible to a higher order two-factor structure, where the care/harm and fairness/cheating foundations comprise the higher order individualizing foundation and the loyalty/betrayal, authority/subversion, and sanctity/degradation foundations comprise the higher order binding foundation (Graham et al., 2011; Nilsson and Erlandsson, 2015). This higher order distinction has also been theoretically brought forward by the developers of MFT (Graham et al., 2009). Other studies were not able to reproduce the original five factor structure of the scale. This seems especially the case for samples from non-WEIRD cultures (Davis et al., 2016; Atari et al., 2020; Iurino and Saucier, 2020; but see Doğruyol et al., 2019 for a contrary result). The scale may be regarded as general in nature, i.e., it does not relate to specific moral issues or behaviors.

For Study 1 and 3, we used six MFQ-items from the relevance part of the questionnaire, measured on a 6-point Likert scale. Three items are related to the care/harm foundation and three to the fairness/cheating foundation, which together make up the higher order individualizing foundation. Factor analysis reveals that the data reproduce this higher order structure as the six items converge on one single factor. The scale was found to be sufficiently reliable (Table 5). Sumscores were computed and the constructed variable representing the individualizing foundation was included as an independent variable in the analyses.

**TABLE 5:** Factor matrix of MFQ-items (studies 1 and 3).

	Questions and items	Factor loadings

**Moral foundations**	**When you decide whether something is right or wrong, to what extent are the following considerations relevant to your thinking? (1 = ‘not at all relevant’ to 6 = ‘extremely relevant’)**	**Individualizing moral foundation** **(α = 0.879)**
Care/harm	Whether or not someone suffered emotionally.	0.667
	Whether or not someone cared for someone weak or vulnerable.	0.755
	Whether or not someone was cruel.	0.762
Fairness/cheating	Whether or not some people were treated differently from others.	0.737
	Whether or not someone acted unfairly.	0.763
	Whether or not someone was denied his or her rights.	0.764

*The analysis was based on the largest sample (N = 2,379, Study 3, see Table 2). Respective analysis on the smaller subsample of Study 1 yielded a similar result. Number of factors extracted was determined based on the factor’s eigenvalues, where the eigenvalue of 1 was used as the cut-off value.*

For Study 2, we used 12 items from the MAC-questionnaire. The MAC-questionnaire is a more recently developed measure of morality. Therefore, it still lacks the track record developed by MFQ in terms of longevity and widespread usage. However, it has been developed as a conceptually as well as psychometrically improved tool with respect to MFQ to measure people’s moral values (Curry et al., 2019). Like MFQ, also MAC-Q can be regarded as general in nature.

The 12 MAC-Q-items that were used in Study 2 belong to four subscales of the judgment part of the questionnaire, with three items for each one. These represent the general moral values of group loyalty, reciprocity, deference, and fairness. The items were measured on a 5-point Likert scale. Factor analysis revealed that our data by and large reproduce the original structure given by MAC. One item (“people have an obligation to help members of their community”) did load higher on a different factor (reciprocity) than it originally belongs to (group loyalty). Still, for considerations with regards to content, we decided to keep it as an item of the group loyalty subscale and preserve the original structure. Reliability analyses showed that the Cronbach Alpha’s of the four subscales consisting of three items each are below the commonly used 0.7 threshold (Table 6). Sumscores were computed of the four subscales and entered as independent variables in the analyses. To check whether the low reliability of the subscales possibly influenced the results, we ran additional regression models in which the 12 MAC-Q-items were included as individual predictors. These models did not lead to substantially different effects than the ones provided in the Results section below. See the section “Discussion” for a further elaboration on this point.

**TABLE 6:** Rotated factor matrix of MAC-Q-items.

Questions and items	Factor loadings			

To what extent do you agree with the following statements? (1 = strongly disagree to 5 = strongly agree)	Group loyalty (α = 0.61)	Reciprocity (α = 0.58)	Deference (α = 0.49)	Fairness (α = 0.60)
People have an obligation to help members of their community.	**0.306**	0.444	0.121	0.158
It’s important for individuals to play an active role in their communities.	**0.638**	0.143	0.110	0.096
You should try to be a useful member of society.	**0.603**	0.212	0.110	0.131
You have an obligation to help those who have helped you	0.131	**0.684**	0.137	0.023
You should always make amends for the things you have done wrong.	0.140	**0.362**	0.184	0.198
You should always return a favor if you can.	0.087	**0.501**	0.174	0.077
People should always defer to their superiors.	–0.022	0.163	**0.562**	–0.038
Society would be better if people were more obedient to authority.	0.134	0.084	**0.510**	–0.028
You should respect people who are older than you.	0.139	0.197	**0.339**	0.075
Everyone should be treated the same	0.018	0.137	0.058	**0.732**
Everyone’s rights are equally important.	0.078	0.130	–0.025	**0.696**
The current levels of inequality in society are unfair.	0.114	0.016	–0.021	**0.366**

*Varimax rotation was used to get a simple structure. Number of factors extracted was determined based on the factor’s eigenvalues, where the eigenvalue of 1 was used as the cut-off value. Loadings in bold signify the item’s selection for the particular factor.*

In Study 3 we considered, besides general moral values, also *specific* moral values as independent variables. Here, we used the previously developed items by de Jonge and van Trijp (2014). These researchers formulated three items to reflect the care/harm and three items to reflect the fairness/cheating foundation, specific to the context of animal welfare. The items are measured on a 7-point Likert scale (totally agree-totally disagree). Factor analysis reveals that all six items converge on one factor, representing the second-order animal specific individualizing moral foundation (Table 7). The scale was found to be sufficiently reliable and the constructed variable was included in the analyses based on sumscores. For the exact description of the items used in the different studies see Tables 5–7.

**TABLE 7:** Factor matrix of the animal-specific moral value items (Study 3).

	Questions and items	Factor loadings

**Animal-specific moral foundations**	**Please indicate to what extent you agree with the following statements (1 = ‘totally disagree’ to 7 = ‘totally agree’)**	**Animal-specific individualizing moral foundation (α = 0.779)**
Care/harm	I don’t care for animal welfare issues. (reverse coded)	0.585
	I feel a strong emotional bond with animals.	0.610
	People exaggerate the feelings and sensitivity of animals. (reverse coded)	0.609
Fairness/cheating	Animals should be protected for their own sake, rather than simply serving the needs of humans.	0.643
	I believe that society has a moral obligation to promote animal welfare.	0.711
	In principle, we as humans have the right to use animals, however, we want to. (reverse coded)	0.520

*Number of factors extracted was determined based on the factor’s eigenvalues, where the eigenvalue of 1 was used as the cut-off value.*

### Social-Demographic Characteristics

Finally, in each model, gender, age, and level of education were included to control for possible spurious effects caused by these social-demographic characteristics. Age was entered as a continuous variable. For Study 1 and 3, gender was entered as a dichotomous dummy variable. Study 2 included a third category for gender, “other,” and therefore two dummy variables were created with male as the reference category to include them in the analysis. For level of education, we created a dichotomous dummy variable and converted all scores to this scale (0 = low/1 = high). Lower education consists of the following levels: primary school; intermediate secondary education; intermediate vocational education. Higher education consists of the levels: higher secondary education; higher vocational education; university.

### Analysis Strategy

To investigate the relationships between the independent and dependent variables we made use of a binary logistic regression analysis and of multiple linear regression analyses. We used the binary logistic regression analysis for the prediction of participation in voluntary behavior (Study 1), as it is operationalized as a dichotomous variable. For the prediction of the other dependent variables, we made use of linear regression models even though most of the dependent variables were measured on Likert scales. While these outcomes are best modeled using ordinal regression models, we relied on the more straightforward linear models, because they are more easily interpreted and because they can provide standardized estimates, indicating the relative importance of the explanatory variables. For our investigation of the relationship between general moral values and behavior we are most interested in these relative effect sizes of our moral predictors. To make sure that our choice for using linear regression models instead of ordinal regression models has not affected our results and conclusions, we ran additional ordinal regression models for each dependent variable. We found that the estimates of the linear regression models (proportionally) match those of the ordinal regression models^13^. We can therefore rely on the presented linear models.

As it is an assumption of factor analysis and PCA, we assume that our variables are normally distributed. A check whether this is the case, indeed showed that the vast majority of the variables approach the normal distribution, with kurtosis and skewness estimates between –1 and 1. As we make use of a large sample (*n* > 1000), the fact that not all variables are normally distributed does not influence our estimates for factor analysis and PCA (Muthén and Kaplan, 1985).

Regarding the linear regression models, for each dependent variable we estimated two consecutive models. In the first, we entered only the social-demographic characteristics, in the second we added the moral value variables to detect the additional effect. All codes of the conducted analyses can be found in the supplementary material.

## Results

In Study 1, we investigated the effects of general moral values on participation in voluntary behavior and providing informal care. Table 8 provides the estimates of the binary logistic regression model, estimating the effects of the social demographic characteristics and the individualizing moral foundation (focusing on care and fairness) on participation in voluntary behavior. The coefficient for the individualizing moral foundation (0.024) is non-significant, providing no evidence that scores on this moral dimension are associated with participation in voluntary behavior. Table 9 presents the standardized coefficients of the linear regression model explaining informal care. Also here (Model 2), the found coefficient for the individualizing moral foundation (0.027) is non-significant. Hence, no evidence is found that people’s scores on this moral dimension influences the extent to which people provide informal care.

**TABLE 8:** Coefficients of the binary logistic regression model predicting voluntary behavior (Study 1).

**Dependent variable:**		
participation in voluntary behavior		

**Independent variables**	**Estimates**	***P-value*** **(two-sided)**

Gender (female)	0.243	0.166
Age	0.015	0.007
Level of education (high)	0.483	0.002
Individualizing moral foundation	0.024	0.142
Constant	–4.309	0.000

**TABLE 9:** Coefficients of regression models predicting voluntary behavior and informal care (Study 1).

	Model 1				Model 2			
Independent variables	Beta	*P-value* (two-sided)	*B*	Standard Error	Beta	*P-value* (two-sided)	*B*	Standard Error
**Dependent variable:** providing informal care								
Gender (female)	0.057	0.006	0.111	0.040	0.055	0.008	0.107	0.040
Age	0.113	0.000	0.007	0.001	0.109	0.000	0.007	0.001
Level of education (high)	–0.013	0.543	–0.023	0.038	–0.017	0.434	–0.030	0.038
Individualizing moral foundation					0.027	0.196	0.005	0.004
*R*-square (sign. change)	0.016	(0.000)			0.017	(0.196)		

*No multicollinearity was found among the independent variables, all VIF values are between 1 and 2.*

Similarly, the results of Study 2 show weak associations between general moral values and moral behaviors (adherence to the Corona measures) (models 4, 5, and 6 of Table 10). Though the effects are somewhat higher than found in Study 1, and several are statistically significant at the 5%-level. The largest two effects, both positive, are the endorsement of the moral value of fairness on adherence to the personal hygiene measures (beta of 0.10) and on adherence to the social distancing measures (beta of 0.15). This means that as one finds fairness more important, one tends to adhere slightly more strictly to the two kinds of corona measures. The moral value of group loyalty has a significant but very weak effect on the adherence to all three forms of corona measures (beta’s of 0.076, 0.061, and 0.067). This suggests that caring about one’s community may play a (small) role in the decision to conform to the imposed measures. The above findings seem intuitive, as both fairness and group loyalty can intelligibly motivate conforming to measures, imposed for the benefit of us all. The very weak, but statistically significant, negative effect for reciprocity on “not visiting the vulnerable” seems less intuitive.

**TABLE 10:** Coefficients of regression models predicting compliance to national Corona measures (Study 2).

	Model 1				Model 4			
Independent variables	Beta	*P-value* (two-sided)	*B*	Standard Error	Beta	*P-value* (two-sided)	*B*	Standard Error
**Dependent variable:** personal hygiene								
Gender (female)	0.219	0.000	0.399	0.047	0.198	0.000	0.363	0.047
Gender (other)	–0.012	0.652	–0.150	0.333	–0.010	0.694	–0.130	0.331
Age	0.172	0.000	0.010	0.002	0.172	0.000	0.010	0.002
Level of education (high)	–0.057	0.031	–0.201	0.093	–0.060	0.024	–0.212	0.094
Group loyalty					0.076	0.009	0.118	0.045
Reciprocity					–0.013	0.644	–0.019	0.041
Deference					0.011	0.689	0.015	0.038
Fairness					0.100	0.000	0.134	0.036
*R*-square (sign. change)	0.089	(0.000)			0.107	(0.000)		

	**Model 2**				**Model 5**			

**Dependent variable:** not visit the vulnerable								
Gender (female)	–0.013	0.613	–0.034	0.068	–0.028	0.289	–0.073	0.069
Gender (other)	–0.024	0.362	–0.438	0.481	–0.025	0.338	–0.458	0.479
Age	–0.168	0.000	–0.014	0.002	–0.177	0.000	–0.015	0.002
Level of education (high)	0.091	0.001	0.452	0.135	0.081	0.003	0.403	0.136
Group loyalty					0.061	0.042	0.133	0.065
Reciprocity					–0.001	0.968	–0.002	0.060
Deference					–0.053	0.062	–0.103	0.055
Fairness					0.082	0.003	0.155	0.052
*R*-square (sign. change)	0.044	(0.000)			0.057	(0.001)		

	**Model 3**				**Model 6**			

**Dependent variable:** social distancing								
Gender (female)	0.130	0.000	0.215	0.043	0.102	0.000	0.168	0.043
Gender (other)	0.057	0.028	0.667	0.303	0.056	0.029	0.654	0.299
Age	0.259	0.000	0.014	0.001	0.256	0.000	0.014	0.001
Level of education (high)	0.092	0.001	0.295	0.085	0.089	0.001	0.285	0.085
Group loyalty					0.067	0.023	0.093	0.041
Reciprocity					–0.065	0.026	–0.083	0.037
Deference					0.013	0.644	0.016	0.035
Fairness					0.147	0.000	0.179	0.033
*R*-square (sign. change)	0.082	(0.000)			0.109	(0.000)		

*No multicollinearity was found among the independent variables, all VIF values are between 1 and 2.*

With 0.1 and 0.15 being the largest effects and the other effects being well under 0.1, the effects should overall be considered as weak. The R-square change of the models 4, 5, and 6 confirm that, although statistically significant, adding the general moral values as predictors to the social-demographics explains only a small additional amount of variance (1.8, 1.3, and 2.7% respectively).

In Study 3, we investigated both the effects of general moral values as well as specific moral values -tailored to the context of animal welfare- on the consumption of meat and meat replacement products. Table 11 (models 3 and 4) shows that the effect of the individualizing moral foundation is non-significant for the frequency of eating chicken meat, and very weak (with a beta of 0.07) for the frequency of eating meat replacement products. Models 5 and 6 show that the context specific individualizing moral foundation (related to animal welfare) is a stronger predictor than its generic counterpart. Its effect on the number of days eating chicken meat is statistically significant at the 5%-level, but is still very weak (beta of –0.07); its effect on eating meat replacement products is substantially stronger than the general moral value (beta of 0.23, significant at 1%-level). Including the specific moral value in model 6 attenuates the initial statistically significant effect of the general moral value on eating meat replacement products (model 4) downwards, rendering it statistically insignificant. This indicates that the initially detected effect of the general moral value is actually explained by its specific counterpart.

**TABLE 11:** Coefficients of regression models predicting consumption of meat and meat replacement products (Study 3).

	Model 1				Model 3				Model 5			
Independent variables	Beta	*P-value* (two-sided)	*B*	Standard Error	Beta	*P-value* (two-sided)	*B*	Standard Error	Beta	*P-value* (two-sided)	*B*	Standard Error
**Dependent variable:** consumption of meat												
Gender (female)	0.017	0.399	0.164	0.194	0.017	0.416	0.158	0.195	0.024	0.246	0.227	0.196
Age	–0.129	0.000	–0.038	0.006	–0.130	0.000	–0.038	0.006	–0.131	0.000	–0.039	0.006
Level of education (high)	–0.007	0.719	–0.066	0.182	–0.009	0.678	–0.076	0.184	–0.009	0.670	–0.078	0.183
Individualizing moral foundation					0.009	0.665	0.008	0.017	0.025	0.247	0.021	0.018
Animal-specific individualizing moral foundation									–0.066	0.002	–0.047	0.015
*R*-square (sign. change)	0.017	0.000			0.017	0.665			0.021	0.002		

	**Model 2**				**Model 4**				**Model 6**			

**Dependent variable:** consumption of meat replacement products												
Gender (female)	0.071	0.000	0.240	0.068	0.067	0.001	0.224	0.068	0.042	0.035	0.140	0.066
Age	–0.037	0.071	–0.004	0.002	–0.046	0.025	–0.005	0.002	–0.042	0.035	–0.004	0.002
Level of education (high)	0.202	0.000	0.628	0.064	0.192	0.000	0.596	0.064	0.192	0.000	0.598	0.062
Individualizing moral foundation					0.074	0.000	0.022	0.006	0.020	0.322	0.006	0.006
Animal-specific individualizing moral foundation									0.225	0.000	0.057	0.005
*R*-square (sign. change)	0.047	0.000			0.052	0.000			0.099	0.000		

*No multicollinearity was found among the independent variables, all VIF values are between 1 and 2.*

In sum, the found effects of general moral values on the considered moral behaviors must be regarded as weak to very weak. Most effects are well under 0.10, while many do not reach statistical significance at the conventional 5%-level. For the models considered, the general moral values are only able to explain 2.7% of variance, at best. Study 3 indicates that context specific values are somewhat stronger predictors than their general counterparts.

## Discussion

Our results show that in all three conducted empirical studies, only weak to very weak effects between general moral values and concrete moral behaviors were found. Overall, the largest effect found within the linear regression models was 0.15, while the large majority of the effects were well under 0.1. Many did not reach statistical significance at the conventional 5%-level. Adding the general moral values as predictors to the models consisting of only the social-demographic characteristics resulted in explaining only very small additional amounts of variance of the behavior. These findings suggest that general moral values are poor predictors of people’s concrete moral behavior.

The findings in Study 3 lend support for the idea that a reason for these weak associations may be sought in the context specificity of moral decision-making. Here, we found larger effects for moral values that were tailored to the context of animal welfare than we found for their generic counterparts. In addition, inclusion of the more specific moral value to the model rendered the (initially found) effect of the general moral value insignificant. This can be explained by the notion that more specific moral values may harbor more accurate information about what is found morally important in a certain kind of context of decision-making, i.e., about how it holds up against factors that may hamper awareness and against other moral and egoistic values that can play a role.

These empirical results are in line with the derived expectations from our conceptual analysis. There, we stated that for a general moral value to act as a predictor of a concrete form of behavior, its presence within an individual would have to (regularly) lead to this behavior. Our conceptual analysis -one we argued to be acceptable to “rationalists” as well as “intuitionists”- suggests that this is unlikely. Before someone’s general moral value can determine behavior in a given situation it needs to go through a process of moral decision-making which consists of four different phases. These all need to become manifest in such a way that they harbor the potential influence of one’s general moral value. When looking into this process, it becomes clear that each phase is influenced by contextual factors, which are not taken into account in a general measurement of moral values and which can potentially annul their influence. We therefore expected to find rather weak effects when predicting concrete moral behavior from general moral values and higher effects for more specific moral values, corresponding to what was found in the empirical studies.

Now the question remains what these results can tell us about predicting concrete moral behavior from general moral values and about the influence of morality on behavior more generally. The results of our empirical study confirm the expectations derived from the conceptual model. They also line up well with previously reported empirical findings on general moral values in relation to moral behavior and with findings from studies on values and behavior more generally. Together, this points toward the notion that general moral values are, in fact, poor predictors of concrete moral behaviors. Our results furthermore suggest that a possible reason for this, is that morality’s influence on behavior may be more context specific than a general questionnaire can grasp. However, we do need to be cautious about drawing too strong conclusions just yet. For our empirical study, we made use of data and data collection methods that were readily available to us. As such, our findings are subject to limitations (but also strengths) that need to be taken into account.

First of all, the samples used in the different studies are not representative of the population. For Study 1 and 3, this may be due to selection effects as the samples were composed of respondents who completed both surveys that were combined for each study. In Study 2, the bias is likely due to the use of convenience sampling. As mentioned in the “Materials and Methods” section, we think this has a limited influence on our results regarding the relationship between general moral values and behavior. The bias most directly affects the estimation of means of the dependent variables, which is not the focus of this study. Though it is possible that the relationship between moral values and behavior for younger or more highly educated people is different than for the Dutch population in general, we have no reason to expect this, also given that no theory of morality argues that morality only influences behavior for select demographic subpopulations.

A strong asset of our study, in terms of preventing bias, is that the measurements for the independent and dependent variables used in Study 1 and 3 (retrieved from the LISS panel data) were collected in different instances with a substantial time in between. This avoids the risk of inducing associations due to the measurements being part of the same survey. For example, if a person just completed a set of items related to general moral values and is then asked whether he or she is engaged in specific forms of moral behavior, the person may be inclined to provide answers that are consistent with his or her stated moral values, thereby inflating the correlations. For study 2, this is a possible limitation as the moral value items and COVID-19 behavior items were part of the same survey. Note, however, that if this has indeed manifested itself, then the actual relationship between moral values and conforming to the considered COVID-19 measures is even weaker than found in our study, which is in support of our main conclusions.

Another limitation of our study is that not all the items of the original scales of MFQ and MAC were available for our analyses, due to using existing data (Study 1 and 3) and space limitations in the conducted survey (Study 2). Specifically, concerning MFQ (Study 1 and 3), we miss measurements for the binding foundations of loyalty, authority, and sanctity; concerning MAC (Study 2) we miss measurements for the values of family, property and heroism. Although we believe that the moral values that were included seem relevant to the behaviors they were to predict (e.g., care with regards to voluntary work and eating meat; deference with regards to compliance behavior with rules), this does limit our empirical findings and corresponding conclusions to the combinations of moral values and behaviors that were studied. Space limitations were also the reason to not include MFQ-items in the survey of Study 2, but limit it to MAC-Q-items.

In addition, for the same reasons as stated above, not all the items per used subscale were available for MFQ and MAC. The latter may be the reason for finding low reliabilities for the MAC’s subscales in our data, which could also be (partly) the reason for finding small effects between these scales and behavior in Study 2. To check whether the outcomes of Study 2 were sensitive to using lesser reliable scales, we ran additional regression analyses including all the 12 MAC-items separately. The results of these analyses provided a similar picture as the results of the analyses based on the MAC-constructs, which were presented in the results section. We found only weak associations and few significant effects between the separate items and the compliance level of the different types of COVID-19-measures.^14^ This result suggests that the found weak effects are not primarily due to the low reliability of the used scales.

Another issue due to using existing data for Study 1 and 3, is that we were somewhat restricted in our choice of the dependent variables. Particularly, though eating chicken meat and eating meat replacement products are instances of (not) eating meat and, therefore, morally relevant behaviors in itself, it would have been more ideal to measure to what extent people (do not) eat meat overall. Especially the eating chicken meat-item is vulnerable to critique in this regard, as eating less chicken meat could also mean that people eat more pork or beef. Also, eating chicken meat may be regarded by some respondents as morally better behavior than eating beef or pork. The weak effect found between the moral value and eating chicken meat may be due to this possible moral ambiguousness of the behavioral item. Whether this is the case can be easily investigated in future research, by using a more encompassing item for measuring individual meat consumption.

A final point concerns the choice of moral behavior more generally. As explained in the “Materials and Methods” section, there may of course be discussion as to what extent these are actually morally relevant behaviors, pertaining to the discussion on imposing morally relevant behavior upon respondents versus having the respondent indicate what he or she deems as moral behavior. There, we substantiated our choice for the first option. However, this does mean that behavior which we have indicated as morally relevant may not be viewed as such by respondents. For instance, “(not) eating meat” or “(not) washing hands during the COVID-19 pandemic” may not be regarded by everybody as morally relevant. Partly, this can be due to not becoming aware that such behavior is actually relevant to one’s moral values. In this case, as explained, these findings are relevant to our conclusions about the relationship between general moral values and behavior. However, if people generally view such behaviors as, for example, health issues, submitted to egoistic considerations rather than moral ones, then our findings say less about the influence of moral values on moral behavior, i.e., we then did not test moral values against genuine moral behavior. As it is possible to question the moral relevance of virtually any behavior to some degree, this seems to be a deeper and almost inevitable issue for the study of moral behavior in general, in particular for studies that choose -for possibly good reasons- to impose what behavior is morally relevant. This dilemma does oblige researchers to sufficiently substantiate this choice, and, in case of imposing the moral behaviors, to substantiate their choices of behavior as well. We hope to have done this to a sufficient degree. Another way to approach this problem is to select a variety of morally relevant behaviors that reflects the rich palette of moral behaviors that exist. We have made an attempt at this in our choices for this study.

In sum, especially the somewhat *ad hoc* character of the selection of moral value- and behavior-items and their accompanying limitations suggests that further research is needed before we can be fully conclusive about whether general moral values are indeed poor predictors of concrete moral behavior in everyday life. Preferably, such research would have to include all and complete subscales and a broad range of selected behaviors. Furthermore, it would be interesting to see whether our findings hold up in more representative samples, as we expect. Another interesting research direction is to use observational data for behavior that is eligible to observe in “real life,” as complementary to self-reports (see e.g., Nilsson et al., 2016; O’Grady et al., 2019 for such efforts).

That said, in light of the fact that our results are in line with studies which have related general moral values to specific forms of moral behavior, as well as with findings from studies on the broader concept of basic values and behavior, and considering that our conceptual framework makes these results intelligible, we do believe that evidence is building up toward the conclusion that general moral values are poor predictors of concrete moral behaviors in daily life. Now let’s assume, for a moment, that we are on the right track with the idea that knowing the moral values that people endorse in general cannot tell us much about their more concrete behaviors. Does this imply that morality or moral values barely influences individual decision-making? And, in its wake, does this mean a blow to the evolutionary foundations and the usefulness of empirical moral value theories? The answer to these questions is left for further research efforts, but we here offer one possible direction.

Our analysis suggests that the contextual aspect of moral decision-making does not square well with the prevailing method used to measure moral values (which is done in a context-free manner through a generic questionnaire, like MFQ and MAC-Q). More specifically, the idea -inevitably assumed by this method - of general moral values being relatively stable personal dispositions that cause a similar kind of behavior over different contexts, does not seem to align with the more dynamic role played by moral values arising from our conception of moral decision-making. In reality, in different specific decision situations a person can become aware of different moral values being at stake, if any; can make different appraisals between moral values as well as between moral values and self-serving considerations; and can make a different assessment of what actions are feasible. This points to a moral influence that is context dependent and possibly much more dynamic than general measurements are able to detect.

Of course, adding more general measures to the model, like people’s moral identity (Aquino and Reed, 2002; Smith et al., 2014), religiosity (Huber and Huber, 2012), or people’s more egoistic values (Seuntjens et al., 2015), may lead to better predictions. This is another aspect that needs to be addressed in further research. However, there are at least two reasons to worry about whether this may solve the indeterminate effect of morality with regards to behavior. First of all, these added variates would again consist of general measures, while things like following through on one’s moral considerations may prove to be rather contextual (Ross and Nisbett, 2011). Secondly, if our elaboration on the process of moral decision-making is on the right track, the indeterminacy seems to be more fundamental. Specifically, in the moral judgment phase, moral values or moral values-inspired considerations are pitted against each other. This seems to constitute an indeterminacy in the heart of the moral endeavor itself. This is no question of whether one is inclined to become aware of the moral relevance of a situation or whether one wants to act morally at all, but how to morally relate to the situation at hand. To get a better understanding of how moral agents weigh considerations within a specific decision situation seems to ask for other measurement techniques in which such weighing has a place.

In line with this, to get a better grasp of the influence of morality on decision-making and action, it is important to know what specific moral values and moral considerations become important within a certain context. This asks for measuring people’s local moral considerations within a certain delineated context or scenario [for an interesting example, see Navarick and Moreno (2022) focusing on people’s moral choices within the delineated setting of COVID-19 triage dilemmas in the hospital]. A further interesting direction in this regard would be to study people’s specific moral considerations across different contexts and see whether and to what extent these, as well as their underlying values, alter per situation.

*In conclusion, the fact that very small effects were found for general moral values and somewhat larger effects for more specific moral values, suggests that people’s moral beliefs can influence decision-making, but that context matters and that this needs to be reflected in the measuring method.* This relates to a fundamental requirement for a valid measurement instrument: it should trigger the same kind of behavioral mechanisms in the measuring process compared to what happens in the real world. To move forward in our understanding of the relationship between morality and behavior this seems crucial. In other words, to know to what extent and how morality influences concrete forms of behavior, such as conforming to COVID-19 measures or eating meat, simply measuring people’s general moral values does not seem the best way to go. Our study suggests that improvement lies in using and developing methods that can better incorporate the contextual aspect of moral decision-making when measuring people’s morality and studying its influence on behavior.

## Data Availability Statement

The datasets presented in this study can be found in online repositories. The names of the repository/repositories and accession number(s) can be found below: for Study 1 and 3 we analyzed existing data collected by CentERdata (Tilburg University, Netherlands) and registered at the LISS panel (www.dataarchive.lissdata.nl). For the sample of Study 1 we combined two surveys: “social integration and leisure, wave 6” (https://www.dataarchive.lissdata.nl/study_units/view/479) and “Consumer heterogeneity with respect to morality in consumption decisions and perceptions of animal welfare – Part 2” (https://www.dataarchive.lissdata.nl/study_units/view/421). For the sample of Study 3 we combined the surveys: “Consumer heterogeneity with respect to morality in consumption decisions and perceptions of animal welfare – Part 1” (https://www.dataarchive.lissdata.nl/study_units/view/420) and “Consumer heterogeneity with respect to morality in consumption decisions and perceptions of animal welfare – Part 2” (https://www.dataarchive.lissdata.nl/study_units/view/421). The dataset that was generated and analyzed for Study 2 is based on our own data collection and is available at the 4tu repository (https://doi.org/10.4121/14242199.v1).

## Ethics Statement

The studies involving human participants were reviewed and approved by the Human Research Ethics Committee of TU Delft. Written informed consent from the participants’ legal guardian/next of kin was not required to participate in this study in accordance with the national legislation and the institutional requirements.

## Author Contributions

TB: conceptualization, methodology, and writing – original draft and editing. MK: conceptualization, methodology, writing – review and editing, and supervision. CC: conceptualization, writing – review and editing, fund acquisition, and supervision. All authors contributed to the article and approved the submitted version.

## Conflict of Interest

The authors declare that the research was conducted in the absence of any commercial or financial relationships that could be construed as a potential conflict of interest.

## Publisher’s Note

All claims expressed in this article are solely those of the authors and do not necessarily represent those of their affiliated organizations, or those of the publisher, the editors and the reviewers. Any product that may be evaluated in this article, or claim that may be made by its manufacturer, is not guaranteed or endorsed by the publisher.

## Footnotes

1 This study focuses on the relationship between general moral values and concrete moral behaviors in everyday life (we use the phrases “everyday life” and “daily life” interchangeably in this paper). By this we mean behaviors that we encounter in, or which are often part of, our everyday endeavors. Unique or heroic deeds are not the focus of this study, neither are behaviors performed in an experimental set-up.

2 In short, “benevolence” is defined as the enhancement of the welfare of people with whom one is in frequent personal contact; “universalism” entails the appreciation and protection of the welfare of all people (Schwartz, 1992).

3 Used behavioral items are for instance: “take it easy and relax,” “do unconventional things,” “make sure everyone I know receives equal treatment” (Bardi and Schwartz, 2003) and “I try to help others” (Caprara et al., 2012).

4 Note that there are also studies investigating the moral value-moral behavior relationship in a game-like or lab settings. As our focus is on the influence of moral values on behavior in everyday life, we leave these studies out of our main reflections. Though these are important studies to investigate certain dynamics of moral behavior (Graham et al., 2012), doubt has been raised to what extent lab findings can be translated to behavior in real life (Levitt and List, 2007; Graham et al., 2012). Either way, findings in these studies do seem to be in line with the above, as only quite weak effects are found (e.g., Schier et al., 2016; Clark et al., 2017; Duc Huynh, 2020).

5 In this study we take context or “contextuality” to come in degrees, moving on a line between the endpoints of “in general” – where there is no context, i.e., there is no articulation of a (kind of) situation as there is, indeed, abstracted from such specificity- to the specific decision situation an individual finds oneself in, on a given moment in time. In between, one can find the broader context in which certain social practices take place. This level has a more general character than the actual decision situation itself, but is more specific than the most abstract endpoint. Think of the context of “driving in traffic,” “the workplace,” or “sustainable food choice”.

6
www.moralfoundations.org

7 Note that we do not necessarily mean *conscious* awareness here. Intuitive or habitual decision-making and behavior may be automatic and stay subconscious. However, this kind of decision-making presupposes some form of (subconscious) recognition of what a certain social situation means. This is here regarded as a form of awareness, albeit subconscious.

8 Interesting in this regard is habitual behavior. It may be argued that habits, harboring a considered general moral value, evade the different phases of the moral decision-making process and thus its indeterminate effects. Here, it seems important to distinguish habitual behavior on, at least, two levels. When we consider a developed moral habit on a somewhat abstract level, such as the habit ‘to help others whenever I can’ -comparable to the moral habits which (neo-) Aristotelians would call moral virtues- the different phases of the moral decision-making model still seem relevant. For instance, one still needs to become aware of situations in which help is needed and, possibly, somehow decide between such tendencies and other habits, like “running away when in danger.” When habitual behavior is considered on a more concrete level, so specific automatic behavior within a particular context, think of “always buying flowers for your neighbor when passing the flower shop on Saturday,” or “always ordering meat when dining out,” this does seem to surpass the indeterminacy of the moral decision-making model. Indeed, developing concrete habitual behavior that harbors an endorsed moral value may then be a way to enhance the congruency between one’s values and behavior. At the same time, it seems that such concrete habitual behavior can also be a source of disconnection between one’s values and behavior. These concrete habits may namely develop in interaction with the social norms within a practical context that do not necessarily reflect one’s own values, e.g., think of the habit of scolding on the referee on a football pitch, while this is not in line with your considered general moral values or behavior in other contexts.

9 Given that we did not gather the data for Study 1 and 3 ourselves, but used existing data, we did not perform power analysis to determine the sample size. The samples that we used for the two studies were constituted by respondents who filled in the two surveys that were combined for each study (see Table 1).

10 Because we gathered the data following a convenience sampling approach in the context of a bachelor course, no power analysis was performed. To gather the data, students invited their social network to fill in the survey. The survey was held open for 2 weeks. The resulting sample was used for the analyses. There were no missing values in the data for all three studies.

11
www.rivm.nl

12 The reason we relied on PCA here and on factor analysis (principal axis factoring) for the MAC-Q-items (and also for the MFQ-items in Study 1 and 3), is that for the latter it is assumed that they are reflective of underlying psychological factors. By applying factor analysis the shared variance among the items is extracted, thereby capturing these psychological factors. The COVID-related behaviors are not thought to be caused by underlying factors. Instead, the PCA is meant to provide summary measures that capture most of the variance in the (behavioral) items (not the shared variance).

13 For reasons of brevity the ordinal regression models are not presented here. The ordinal regression models can be found in the Supplementary Material of this article.

14 These results are not reported here, for brevity, but are published as Supplementary Material to this article.
