# Supplementary material for: Why Are General Moral Values Poor Predictors of Concrete Moral Behavior in Everyday Life? A Conceptual Analysis and Empirical Study
Source: Front Psychol. 2022 Jun 30;13:817860. doi: 10.3389/fpsyg.2022.817860 (PMC9377516; doi:10.3389/fpsyg.2022.817860)
Supplement: Supplementary file 2 [file Table_2.docx]

Description and codes of the performed statistical analyses belonging to the paper ‘Why are general moral values poor predictors of concrete moral behavior in everyday life? a conceptual analysis and empirical study’

Study 1

For study 1 and 3 we analysed existing data collected by CentERdata (Tilburg University, Netherlands) and registered at the LISS panel (www.dataarchive.lissdata.nl)

**File:** For the sample of study 1 we combined two surveys: ‘social integration and leisure, wave 6' (https://www.dataarchive.lissdata.nl/study_units/view/479) and 'Consumer heterogeneity with respect to morality in consumption decisions and perceptions of animal welfare - Part 2' (https://www.dataarchive.lissdata.nl/study_units/view/421).

This yielded a sample of 2,320

**Data preparation**

*Independent variables*

To investigate the structure of the data with regards to the independent variables we performed factor analysis on the MFQ-items (hr12a078 to hr12a083)

Code:

FACTOR

/VARIABLES hr12a078 hr12a079 hr12a080 hr12a081 hr12a082 hr12a083

/MISSING LISTWISE

/ANALYSIS hr12a078 hr12a079 hr12a080 hr12a081 hr12a082 hr12a083

/PRINT INITIAL EXTRACTION

/FORMAT BLANK(.30)

/CRITERIA MINEIGEN(1) ITERATE(25)

/EXTRACTION PAF

/ROTATION NOROTATE

/METHOD=CORRELATION.

This produced a 1-factor structure, replicating the original higher level individualizing foundation factor. A new variable was created by computing the sumscore of the 6-items and called it ‘individualizing’

*Dependent variables:*

The variable representing whether respondents participated in voluntary work ‘voluntary_behv_binary’ was constituted by recoding three items asking about participation in a particular field of voluntary work (Religious, environmental, and human rights) into a dichotomous variable (0=no/1=yes).

The variable representing the amount of informal care that people provided was constituted by recoding the reported amount of hours into 6 categories (see Table 7 for the distribution). This category was called ‘caredays’.

*Social demographic characteristics*

Age was entered in the analyses as a continuous variable called ‘leeftijd’.

For gender there were three categories in the survey: male, female, and other. Therefore, two dummy variables were created with male as reference category: ‘Geslacht_dummy_vrouw’ (female) and ‘Geslacht_dummy_Anders’ (other).

For level of education, we created a dichotomous dummy variable ‘Dummy3_opleiding_MBO4laag’ and converted all scores to this scale (0=low/ 1=high). Lower education consists of the following levels: primary school; intermediate secondary education, intermediate vocational education (categories 1 to 5). Higher education consists of the levels: higher secondary education; higher vocational education; university (categories 6 to 8).

**Multiple regression analyses**

To explore the relationship between the independent and dependent variables, we made use of binary logistic regression analysis to predict participation in voluntary behavior and linear regression analysis to predict the level of provided informal care.

*Binary logistic regression analysis -dependent variable: voluntary_behv_binary*

Code:

LOGISTIC REGRESSION VARIABLES voluntary_behv_binary

/METHOD=ENTER Gender_dummy leeftijd Educ_dummy_MBOlaag individualizing

/CRITERIA=PIN(.05) POUT(.10) ITERATE(20) CUT(.5).

*Linear regression analysis- dependent variable: caredays*

Code:

REGRESSION

/MISSING LISTWISE

/STATISTICS COEFF OUTS R ANOVA COLLIN TOL CHANGE

/CRITERIA=PIN(.05) POUT(.10)

/NOORIGIN

/DEPENDENT caredays

/METHOD=ENTER Gender_dummy leeftijd Educ_dummy_MBOlaag individualizing

/RESIDUALS DURBIN.

Study 2

**File:** The dataset that was generated and analysed for study 2 is available in the 4tu repository (https://doi.org/10.4121/14242199.v1)

FILE='H:\Project\psychometrics\paper 2\Data corona\Data2_general moral values _coronabehavior_Study2_PHB_4turep_Data corona 19 juni geaggregeerd.sav'.

**Data preparation**

*Independent variables*

To investigate the structure of the data with regards to the *independent variables* we performed factor analysis on the MAC-items (intrinsiek 1 to intrinsiek 12), using Varimax rotation.

Code:

FACTOR

/VARIABLES intrinsiek_1 intrinsiek_2 intrinsiek_3 intrinsiek_4 intrinsiek_5 intrinsiek_6

intrinsiek_7 intrinsiek_8 intrinsiek_9 intrinsiek_10 intrinsiek_11 intrinsiek_12

/MISSING LISTWISE

/ANALYSIS intrinsiek_1 intrinsiek_2 intrinsiek_3 intrinsiek_4 intrinsiek_5 intrinsiek_6

intrinsiek_7 intrinsiek_8 intrinsiek_9 intrinsiek_10 intrinsiek_11 intrinsiek_12

/PRINT INITIAL EXTRACTION ROTATION

/FORMAT BLANK(.30)

/CRITERIA MINEIGEN(1) ITERATE(25)

/EXTRACTION PAF

/CRITERIA ITERATE(25)

/ROTATION VARIMAX

/METHOD=CORRELATION.

This produced a 4-factor solution largely replicating the factors given by the original structure of the MAC-scale (Group Loyalty, Reciprocity, Deference, and Fairness). As explained in the paper, one item (‘intrinsiek 1’) loads higher on a different factor (reciprocity) than it originally belongs to (group loyalty). Still, for considerations with regards to content, we decided to keep it as an item of the group loyalty subscale and preserve the original structure of the scale.

We created new variables by computing the sumscores for the items belonging to each factor (see Table 4 in the paper which item belongs to which factor). These were named ‘Belang_van_groepswaarden’ (group loyalty), ‘Belang_van_wederkerigheid’ (reciprocity), ‘Belang_van_eerbied’ (deference), and ‘Belang_van_gelijkheid’ (fairness).

*Dependent variables*

To effectively summarize the data and reduce the number of dependent variables we performed a PCA on the corona-measure items (regels_1 to regels_10), using Varimax rotation. This resulted in a solution where one item did not clearly load on one of the given components (regels_5). Also contentwise it seemed acceptable to leave this item out of further analysis (about sneezing and coughing in the elbow). We again performed a PCA, using Varimax rotation on the corona-measure items except for regels_5

Code:

FACTOR

/VARIABLES regels_1 regels_2 regels_3 regels_4 regels_6 regels_7 regels_8 regels_9 regels_10

/MISSING LISTWISE

/ANALYSIS regels_1 regels_2 regels_3 regels_4 regels_6 regels_7 regels_8 regels_9 regels_10

/PRINT INITIAL EXTRACTION ROTATION

/FORMAT BLANK(.30)

/CRITERIA MINEIGEN(1) ITERATE(25)

/EXTRACTION PC

/CRITERIA ITERATE(25)

/ROTATION VARIMAX

/METHOD=CORRELATION.

This produced a 3-component structure that was intelligibly interpretable. We created new variables by computing the sumscore for each component (see Table 6 in the paper which item belongs to which component) and named these constructed variables: ‘SOM_pershygiene’ (personal hygiene), ‘SOM_afstandZwakke’ (not visit the vulnerable), ‘SOM_socialdistancing’ (social distancing).

*Social demographic characteristics*

Age was entered in the analyses as a continuous variable called ‘leeftijd’.

For gender there were three categories in the survey: male, female, and other. Therefore, two dummy variables were created with male as reference category: ‘Geslacht_dummy_vrouw’ (female) and ‘Geslacht_dummy_Anders’ (other).

For level of education, we created a dichotomous dummy variable ‘Dummy3_opleiding_MBO4laag’ and converted all scores to this scale (0=low/ 1=high). Lower education consists of the following levels: primary school; intermediate secondary education, intermediate vocational education (categories 1 to 5). Higher education consists of the levels: higher secondary education; higher vocational education; university (categories 6 to 8).

**Multiple Regression Analyses**

To explore the relationships between the independent and dependent variables we made use of linear regression analyses. For each dependent variable we estimated two consecutive models. In the first we entered only the social-demographic characteristics, in the second we added the moral value variables to detect the additional effect.

*Dependent variable: SOM_pershygiene (personal hygiene) Table 9 (model 1 and 4) in paper*

Code:

REGRESSION

/MISSING LISTWISE

/STATISTICS COEFF OUTS R ANOVA COLLIN TOL CHANGE

/CRITERIA=PIN(.05) POUT(.10)

/NOORIGIN

/DEPENDENT SOM_pershygiene

/METHOD=ENTER Geslacht_dummy_vrouw Geslacht_dummy_Anders leeftijd Dummy3_opleiding_MBO4laag

/METHOD=ENTER Belang_van_groepswaarden Belang_van_wederkerigheid Belang_van_eerbied

Belang_van_gelijkheid

/RESIDUALS DURBIN.

*Dependent variable: SOM_afstandZwakke (not visit the vulnerable), Table 9 (Model 2 and 5) in paper*

Code:

REGRESSION

/MISSING LISTWISE

/STATISTICS COEFF OUTS R ANOVA

/CRITERIA=PIN(.05) POUT(.10)

/NOORIGIN

/DEPENDENT SOM_afstandZwakke

/METHOD=ENTER Geslacht_dummy_vrouw Geslacht_dummy_Anders leeftijd Dummy3_opleiding_MBO4laag

/METHOD=ENTER Belang_van_groepswaarden Belang_van_wederkerigheid Belang_van_eerbied

Belang_van_gelijkheid.

*Dependent variable: SOM_socialdistancing (social distancing) Table 9 (Model 3 and 6) in paper*

Code:

REGRESSION

/MISSING LISTWISE

/STATISTICS COEFF OUTS R ANOVA

/CRITERIA=PIN(.05) POUT(.10)

/NOORIGIN

/DEPENDENT SOM_socialdistancing

/METHOD=ENTER Geslacht_dummy_vrouw Geslacht_dummy_Anders leeftijd Dummy3_opleiding_MBO4laag

/METHOD=ENTER Belang_van_groepswaarden Belang_van_wederkerigheid Belang_van_eerbied

Belang_van_gelijkheid.

Study 3

For study 1 and 3 we analysed existing data collected by CentERdata (Tilburg University, Netherlands) and registered at the LISS panel (www.dataarchive.lissdata.nl)

For the sample of study 3 we combined two surveys: 'Consumer heterogeneity with respect to morality in consumption decisions and perceptions of animal welfare - Part 1' (https://www.dataarchive.lissdata.nl/study_units/view/420) and 'Consumer heterogeneity with respect to morality in consumption decisions and perceptions of animal welfare - Part 2' (<https://www.dataarchive.lissdata.nl/study_units/view/421>).

This yielded a sample of: 2,379

**Data preparation**

*Independent variables*

To investigate the structure of the data with regards to the MFQ-items (hr12a078 to hr12a083) we performed factor analysis (hr12a078 to hr12a083)

Code:

FACTOR

/VARIABLES hr12a078 hr12a079 hr12a080 hr12a081 hr12a082 hr12a083

/MISSING LISTWISE

/ANALYSIS hr12a078 hr12a079 hr12a080 hr12a081 hr12a082 hr12a083

/PRINT INITIAL EXTRACTION

/FORMAT BLANK(.30)

/CRITERIA MINEIGEN(1) ITERATE(25)

/EXTRACTION PAF

/ROTATION NOROTATE

/METHOD=CORRELATION.

This produced a 1-factor structure, replicating the original higher level individualizing foundation factor. A new variable was created by computing the sumscore of the 6 items and called it ‘individualizing’

To investigate the structure of the data with regards to the animal specific moral value-items (hr12a099 to hr12a104) we performed a factor analysis

Code:

FACTOR

/VARIABLES hr12a099 hr12a100 hr12a101 hr12a102 hr12a103 hr12a104

/MISSING LISTWISE

/ANALYSIS hr12a099 hr12a100 hr12a101 hr12a102 hr12a103 hr12a104

/PRINT INITIAL EXTRACTION

/FORMAT BLANK(.30)

/CRITERIA MINEIGEN(1) ITERATE(25)

/EXTRACTION PAF

/ROTATION NOROTATE

/METHOD=CORRELATION.

This produced a 1-factor structure. We created a new variable by computing the sumscore of the 6 items and called it ‘animal_individualizing’

*Dependent variables*

As dependent variables we used variable ‘hq12a072’ (In a period of 4 weeks (28 days), how many days do you eat the below mentioned meats? Chicken meat) for the consumption of chicken meat.

And the variable ‘meatreplace’ (Do you ever eat meat replacement products? By meat replacements products we mean vegetarian balls or burgers (for example from Vivera, Goodbite, Tivall, Valess), tofu, soy, tempé, or quorn) for eating meat replacement products.

*Social demographic characteristics*

Age was entered in the analyses as a continuous variable called ‘leeftijd’.

For gender a dummy variable was created with male as reference category: ‘Gender_dummy’

For level of education, we created a dichotomous dummy variable ‘Educ_dummy_MBOlaag’ and converted all scores to this scale (0=low/ 1=high). Lower education consists of the following levels: primary school; intermediate secondary education, intermediate vocational education (categories 1,2 and 4). Higher education consists of the levels: higher secondary education; higher vocational education; university (categories 3, 5, and 6).

**Multiple regression analysis**

To explore the relationships between the independent and dependent variables we made use of linear regression analyses. For each dependent variable we estimated three consecutive models. In the first we entered only the social-demographic characteristics, in the second we added the general moral value variable (‘individualizing’) and in the third also the specific moral value variable (‘animal_individualizing’) to detect the additional effects.

*Dependent variable: ‘hq12a072’ (consumption of chicken meat) Table 10 (Model 1, 3, and 5) in paper*

Code:

REGRESSION

/MISSING LISTWISE

/STATISTICS COEFF OUTS R ANOVA COLLIN TOL CHANGE

/CRITERIA=PIN(.05) POUT(.10)

/NOORIGIN

/DEPENDENT hq12a072

/METHOD=ENTER Gender_dummy leeftijd Educ_dummy_MBOlaag

/METHOD=ENTER individualizing

/METHOD=ENTER animal_individualizing

/RESIDUALS DURBIN.

*Dependent variable: ‘meatreplace’ (concumption of meatreplacement products) Table 10 (model 2, 4, and 6) in paper*

Code:

REGRESSION

/MISSING LISTWISE

/STATISTICS COEFF OUTS R ANOVA COLLIN TOL CHANGE

/CRITERIA=PIN(.05) POUT(.10)

/NOORIGIN

/DEPENDENT meatreplace

/METHOD=ENTER Gender_dummy leeftijd Educ_dummy_MBOlaag

/METHOD=ENTER individualizing

/METHOD=ENTER animal_individualizing

/RESIDUALS DURBIN.

**Codes of additional regression models in supplementary material**

Ordinal regression models

**Study 1**

*Dependent variable: voluntary behavior*

Code:

PLUM voluntary_behv_binary WITH Gender_dummy leeftijd Educ_dummy_MBOlaag individualizing

/CRITERIA=CIN(95) DELTA(0) LCONVERGE(0) MXITER(100) MXSTEP(5) PCONVERGE(1.0E-6) SINGULAR(1.0E-8)

/LINK=LOGIT

/PRINT=FIT PARAMETER SUMMARY.

*Dependent variable: providing informal care*

Code:

PLUM caredays WITH Gender_dummy leeftijd Educ_dummy_MBOlaag individualizing

/CRITERIA=CIN(95) DELTA(0) LCONVERGE(0) MXITER(100) MXSTEP(5) PCONVERGE(1.0E-6) SINGULAR(1.0E-8)

/LINK=LOGIT

/PRINT=FIT PARAMETER SUMMARY.

**Study 2**

*Dependent variable: Personal hygiene*

PLUM SOM_pershygiene WITH Geslacht_dummy_vrouw Geslacht_dummy_Anders leeftijd

Dummy3_opleiding_MBO4laag Belang_van_groepswaarden Belang_van_wederkerigheid Belang_van_eerbied

Belang_van_gelijkheid

/CRITERIA=CIN(95) DELTA(0) LCONVERGE(0) MXITER(100) MXSTEP(5) PCONVERGE(1.0E-6) SINGULAR(1.0E-8)

/LINK=LOGIT

/PRINT=FIT PARAMETER SUMMARY.

*Dependent variable: not visit the vulnerable*

PLUM SOM_afstandZwakke WITH Geslacht_dummy_vrouw Geslacht_dummy_Anders leeftijd

Dummy3_opleiding_MBO4laag Belang_van_groepswaarden Belang_van_wederkerigheid Belang_van_eerbied

Belang_van_gelijkheid

/CRITERIA=CIN(95) DELTA(0) LCONVERGE(0) MXITER(100) MXSTEP(5) PCONVERGE(1.0E-6) SINGULAR(1.0E-8)

/LINK=LOGIT

/PRINT=FIT PARAMETER SUMMARY.

*Dependent variable: social distancing*

PLUM SOM_socialdistancing WITH Geslacht_dummy_vrouw Geslacht_dummy_Anders leeftijd

Dummy3_opleiding_MBO4laag Belang_van_groepswaarden Belang_van_wederkerigheid Belang_van_eerbied

Belang_van_gelijkheid

/CRITERIA=CIN(95) DELTA(0) LCONVERGE(0) MXITER(100) MXSTEP(5) PCONVERGE(1.0E-6) SINGULAR(1.0E-8)

/LINK=LOGIT

/PRINT=FIT PARAMETER SUMMARY.

**Study 3**

*Dependent variable: eating chicken meat*

Code:

PLUM hq12a072 WITH Gender_dummy leeftijd Educ_dummy_MBOlaag individualizing animal_individualizing

/CRITERIA=CIN(95) DELTA(0) LCONVERGE(0) MXITER(100) MXSTEP(5) PCONVERGE(1.0E-6) SINGULAR(1.0E-8)

/LINK=LOGIT

/PRINT=FIT PARAMETER SUMMARY.

*Dependent variable: eating meat replacement products*

Code:

PLUM meatreplace WITH Gender_dummy leeftijd Educ_dummy_MBOlaag individualizing

animal_individualizing

/CRITERIA=CIN(95) DELTA(0) LCONVERGE(0) MXITER(100) MXSTEP(5) PCONVERGE(1.0E-6) SINGULAR(1.0E-8)

/LINK=LOGIT

/PRINT=FIT PARAMETER SUMMARY.

Study 2 -linear regression models with the 12 MAC-items as separate predictors

*Dependent variable: Personal hygiene*

Code:

REGRESSION

/MISSING LISTWISE

/STATISTICS COEFF OUTS R ANOVA COLLIN TOL CHANGE

/CRITERIA=PIN(.05) POUT(.10)

/NOORIGIN

/DEPENDENT SOM_pershygiene

/METHOD=ENTER Geslacht_dummy_vrouw Geslacht_dummy_Anders leeftijd Dummy3_opleiding_MBO4laag

intrinsiek_1 intrinsiek_2 intrinsiek_3 intrinsiek_4 intrinsiek_5 intrinsiek_6 intrinsiek_7

intrinsiek_8 intrinsiek_9 intrinsiek_10 intrinsiek_11 intrinsiek_12

/RESIDUALS DURBIN.

*Dependent variable: Distance vulnerable*

Code:

REGRESSION

/MISSING LISTWISE

/STATISTICS COEFF OUTS R ANOVA COLLIN TOL CHANGE

/CRITERIA=PIN(.05) POUT(.10)

/NOORIGIN

/DEPENDENT SOM_afstandZwakke

/METHOD=ENTER Geslacht_dummy_vrouw Geslacht_dummy_Anders leeftijd Dummy3_opleiding_MBO4laag

intrinsiek_1 intrinsiek_2 intrinsiek_3 intrinsiek_4 intrinsiek_5 intrinsiek_6 intrinsiek_7

intrinsiek_8 intrinsiek_9 intrinsiek_10 intrinsiek_11 intrinsiek_12

/RESIDUALS DURBIN.

*Dependent variable: social distancing*

Code:

REGRESSION

/MISSING LISTWISE

/STATISTICS COEFF OUTS R ANOVA COLLIN TOL CHANGE

/CRITERIA=PIN(.05) POUT(.10)

/NOORIGIN

/DEPENDENT SOM_socialdistancing

/METHOD=ENTER Geslacht_dummy_vrouw Geslacht_dummy_Anders leeftijd Dummy3_opleiding_MBO4laag

intrinsiek_1 intrinsiek_2 intrinsiek_3 intrinsiek_4 intrinsiek_5 intrinsiek_6 intrinsiek_7

intrinsiek_8 intrinsiek_9 intrinsiek_10 intrinsiek_11 intrinsiek_12

/RESIDUALS DURBIN.
